# A QSAR, Pharmacokinetic and Toxicological Study of New Artemisinin Compounds with Anticancer Activity

**DOI:** 10.3390/molecules190810670

**Published:** 2014-07-24

**Authors:** Josinete B. Vieira, Francinaldo S. Braga, Cleison C. Lobato, César F. Santos, Josivan S. Costa, José Adolfo H. M. Bittencourt, Davi S. B. Brasil, Jocivânia O. Silva, Lorane I. S. Hage-Melim, Williams Jorge C. Macêdo, José Carlos T. Carvalho, Cleydson Breno R. Santos

**Affiliations:** 1 Laboratory of Modeling and Computational Chemistry, Federal University of Amapá, Rod JK Km2, Macapá, Amapá 68902-280, Brazil; E-Mails: jnetbio.unifap2011.ap@gmail.com (J.B.V.); fsbraga@unifap.br (F.S.B.); cleyson.cl@gmail.com (C.C.L.); cesar.fs@globomail.com (C.F.S.); josivan.chemistry@gmail.com (J.S.C.); davibb@ufpa.br (D.S.B.B.); lorane@unifap.br (L.I.S.H.-M.); williams.macedo@ufra.edu.br (W.J.C.M.); 2 Postgraduate Program in Pharmaceutical Sciences, Federal University of Amapá, Rod JK Km 2, Macapá, Amapá 68902-280, Brazil; E-Mails: jocivania@unifap.br (J.O.S.); farmacos@unifap.br (J.C.T.C.); 3 Laboratory of Drug Research, School of Pharmaceutical Sciences, Federal University of Amapá, Macapá, Amapá 68902-280, Brazil; E-Mail: joseadolfo@unifap.br; 4 Institute of Technology, Federal University of Pará, Av. Augusto Corrêa, 01, Belém, Pará 66075-900, Brazil; 5 Laboratory of Molecular Modeling and Simulation System, Federal Rural University of Amazônia, Rua João Pessoa, 121, Campus Capanema-Centro, Capanema, Pará 68700-030, Brazil

**Keywords:** artemisinin, anticancer activity, molecular modeling, B3LYP/6-31G**, QSAR

## Abstract

The Density Functional Theory (DFT) method and the 6-31G** basis set were employed to calculate the molecular properties of artemisinin and 20 derivatives with different degrees of cytotoxicity against the human hepatocellular carcinoma HepG2 line. Principal component analysis (PCA) and hierarchical cluster analysis (HCA) were employed to select the most important descriptors related to anticancer activity. The significant molecular descriptors related to the compounds with anticancer activity were the ALOGPS_log, Mor29m, IC5 and GAP energy. The Pearson correlation between activity and most important descriptors were used for the regression partial least squares (PLS) and principal component regression (PCR) models built. The regression PLS and PCR were very close, with variation between PLS and PCR of R^2^ = ±0.0106, R^2^_ajust_ = ±0.0125, s = ±0.0234, F_(4,11)_ = ±12.7802, Q^2^ = ±0.0088, SEV = ±0.0132, PRESS = ±0.4808 and S_PRESS_ = ±0.0057. These models were used to predict the anticancer activity of eight new artemisinin compounds (test set) with unknown activity, and for these new compounds were predicted pharmacokinetic properties: human intestinal absorption (HIA), cellular permeability (P_CaCO2_), cell permeability Maden Darby Canine Kidney (P_MDCK_), skin permeability (P_Skin_), plasma protein binding (PPB) and penetration of the blood-brain barrier (C_Brain/Blood_), and toxicological: mutagenicity and carcinogenicity. The test set showed for two new artemisinin compounds satisfactory results for anticancer activity and pharmacokinetic and toxicological properties. Consequently, further studies need be done to evaluate the different proposals as well as their actions, toxicity, and potential use for treatment of cancers.

## 1. Introduction

Cancer, also called malignant neoplasm or malignant tumor, is a disease characterized by the uncontrolled growth of abnormal cells in an organism [1]. While the origin of these is due to genetic alterations may be by inactivation of tumor suppressor genes, activation of oncogenes, inactivation of genes responsible for apoptosis and mutations produced by chemical, physical and biological agents, and are characterized by loss of function coming from the absence of differentiation, uncontrolled proliferation, invasiveness of adjacent tissues and metastasis [2,3].

On a global scale there was an increase to 14.1 million new cases of different types of cancer in 2012, causing 8.2 million deaths, in accordance with the online channel GLOBOCAN 2012 [4]. The prevalence estimates for 2012 show that there were 32.6 million people (over the age of 15 years) who have had a cancer diagnosed in the last five years. The types most commonly diagnosed around the world were lung (1.8 million, 13.0% of the total), breast (1.7 million, 11.9%), and colon and rectum (1.4 million, 9.7%). The most common determinants of death were lung cancers (1.6 million, 19.4% of the total), liver (0.8 million, 9.1%) and stomach (0.7 million, 8.8%). Importantly, among the different forms of cancer malignant tumors of the liver, hepatocellular carcinoma type, is the second most common causing deaths around the world [5].

Nowadays a variety of factors has driven the search for new drugs of plant origin, particularly the discovery of drugs that fight cancer effectively [6]. Chaturvedi [7] relates that nowadays the antitumor action is the most widely studied biological activity of sesquiterpene lactones, where studies reveal that these are capable of combating tumors via selective alkylation, thereby controlling and inhibiting cell division. This set of factors and cellular functions leads the cells to lose action by apoptosis.

There are some drugs derived from sesquiterpene lactones such as artemisinin, that in clinical trials showed activity to combat cancer [7,8,9]. *Artemisia annua* L. a plant species coming from temperate regions such as China and Southeast Europe, contains the active principle artemisinin (qinghaosu), that is widely used in traditional Chinese medicine for the treatment of malaria [10].

**Figure 1:**
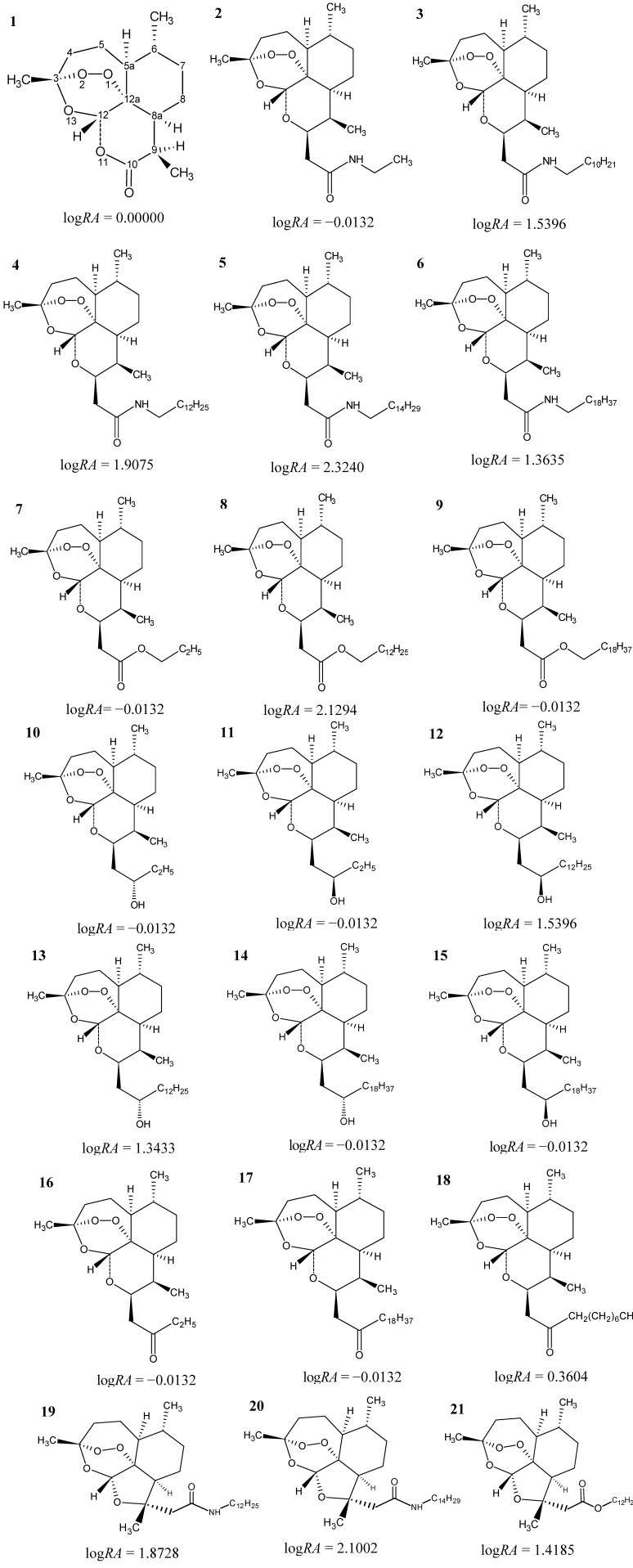
Structure and biological activity of artemisinin and derivatives with anticancer activity against human hepatocellular carcinoma HepG2 line.

Recently artemisinin (Figure 1, compound 1) has been reported for its ability to exert a cytotoxic effect on cancer cells [11]. Studies of the activity of artemisinin and its derivatives appear to indicate it is mediated by its interaction through the endoperoxide function of the 1,2,13-trioxane ring [12]. Therefore, it becomes necessary to discover the mechanism of action of the compound to be studied in order to determine how to carry out drug-receptor interactions, for this is necessary the utilization of some tools such as the use of molecular modeling that enables one to determine cell sites or the physiology involved in this process [13].

Molecular modeling is a tool that consists in the application of theoretical models to represent and manipulate the structure of molecules, study chemical reactions and establish relationships between structure and properties of matter [14,15]. In the theoretical chemistry area there are some strategies that are promising in relation to the design of new drugs, such as rational design, which consists of using information in different areas of human knowledge, especially those related to the electronic levels of the drug, physical-chemical parameters (hydrophobic, steric and electronic) related with the biological activity [16,17,18,19]. This type of strategy, unlike molecular modification, does not have high time demands and is low in financial investment. Among the various techniques we can highlight planning with the help of computer, which is a resource that increases considerably the possibilities of scientific research in discovery of new drugs [20,21,22,23].

In this paper, a QSAR study of artemisinin and 20 derivatives with logarithm of relative activity, log*RA* (see Figure 1) that showed different degrees of cytotoxicity against the human hepatocellular carcinoma HepG2 line [24]. Initially, the structures were modeled, and many different molecular descriptors were computed. Principal Component Analysis (PCA) and Hierarchical Cluster Analysis (HCA) were employed to choose the molecular descriptors that are most related to the anticancer biological property investigated. Then, a QSAR model was elaborated through the Principal Component Regression (PCR) and Partial Least Square (PLS) methods that were used to perform predictions for eight new artemisinin compounds (test set) with unknown anticancer activity [25,26,27,28]. For these eight compounds the following pharmacokinetic properties: human intestinal absorption (HIA), cellular permeability (P_CaCO2_), cell permeability Maden Darby Canine Kidney (P_MDCK_), skin permeability (P_Skin_), plasma protein binding (PPB) and penetration of the blood-brain barrier (C_Brain/Blood_), and toxicological ones, mutagenicity and carcinogenicity, were predicted. These predictions aid in the interactions between micromolecules and their molecular targets, predicting, also, possible toxic consequences of the drug candidate and to aid in future studies searching for other new anticancer drugs.

## 2. Results and Discussion

### 2.1. Determination of the Theoretical Geometrical Parameters for the 1,2,13-Trioxane Ring of Artemisinin (Bond Length, Bond Angle, and Torsion Angle of Atoms in this Ring) in Different Methods and Basis Sets

We determined the geometrical parameters for the 1,2,13-trioxane ring of artemisinin (bond length, bond angle, and torsion angle of atoms in this ring), as shown in Table 1.

**Table 1:** Theoretical and experimental parameters for the 1,2,13-trioxane ring in artemisinin (compound 1).

Parameters ^[a]^	Semiempirical			Hartree-Fock/HF							DFT/B3LYP				EXP [29]
	AM1	PM3	ZINDO	6-31G	6-31G*	6-31G**	3-21G	3-21G*	3-21G**	6-311G	6-31G	6-31G*	6-31G** ^[b]^	3-21G	
Bond length (Å)															
O1O2	1.288	1.544	1.237	1.447	1.391	1.390	1.461	1.461	1.462	1.429	1.524	1.459	1.459	1.524	1.469
O2C3	1.447	1.403	1.400	1.435	1.393	1.396	1.440	1.440	1.439	1.432	1.452	1.413	1.414	1.455	1.416
C3O13	1.427	1.428	1.396	1.435	1.388	1.408	1.436	1.435	1.435	1.434	1.473	1.441	1.441	1.473	1.445
O13C12	1.416	1.403	1.392	1.403	1.400	1.376	1.407	1.407	1.407	1.401	1.425	1.395	1.396	1.430	1.379
C12C12a	1.537	1.555	1.513	1.533	1.533	1.532	1.529	1.529	1.530	1.530	1.438	1.539	1.539	1.535	1.523
C12aO1	1.468	1.427	1.416	1.469	1.429	1.429	1.477	1.477	1.477	1.438	1.499	1.455	1.455	1.504	1.461
Bond angle (°)															
O1O2C3	112.530	110.340	114.310	108.800	106.100	109.460	107.100	107.080	107.060	109.210	107.300	108.280	108.280	105.590	108.100
O2C3O13	103.600	104.810	105.370	106.760	110.800	107.800	107.270	107.285	107.300	106.670	107.730	108.490	108.490	108.220	106.600
C3O13C12	115.480	116.010	115.843	117.300	112.800	115.300	115.670	115.680	115.710	116.960	114.990	114.080	114.060	113.200	114.200
O13C12C12a	113.510	115.200	113.270	112.280	108.700	112.300	112.080	112.080	112.030	112.360	113.640	113.250	113.240	113.300	114.500
C12C12aO1	111.070	113.180	107.290	110.910	110.500	110.545	111.570	111.600	111.600	110.760	111.740	111.290	111.280	112.410	110.700
C12aO1O2	113.740	112.290	118.380	113.240	112.700	112.700	111.290	111.290	111.290	113.360	111.400	111.600	111.590	109.620	111.200
Torsion angle (°)															
O1O2C3O13	−77.800	−73.310	−70.403	−71.840	−73.369	−73.400	−74.670	−74.700	−74.690	−71.940	−73.460	−73.900	−73.910	−76.610	−75.500
O2C3O13C12	42.070	52.700	36.370	33.390	31.034	31.100	32.300	32.360	32.180	33.010	34.970	32.800	32.780	33.750	36.000
C3O13C12C12a	11.400	2.811	17.420	25.320	27.432	27.400	28.290	28.190	28.330	25.380	26.260	27.460	25.500	29.060	25.300
O13C12C12aO1	−41.770	−40.510	−46.610	−49.410	−50.100	−50.143	−50.860	−50.770	−50.700	−49.470	−51.200	−51.270	−51.340	−52.190	−51.300
C12C12aO1O2	12.050	19.940	18.110	12.510	10.900	10.924	9.989	9.940	9.750	12.480	12.740	11.730	11.780	9.060	12.700
C12aO1O2C3	47.050	35.630	40.130	46.700	48.700	48.674	50.330	50.350	50.530	46.870	46.900	47.850	47.830	51.060	47.800
Standard Deviation	4.776	8.388	4.372	1.663	2.484	1.762	1.722	1.714	1.797	1.658	0.843	1.227	1.103	1.915	˗

^[a]^: The atoms are numbered according to compound 1 in Figure 1; ^[b]^: Valence basis set separately validated for calculating the molecular properties.

Table 1 illustrates that for the DFT method, all four basis sets (B3LYP/6-31G, B3LYP/6-31G*, B3LYP/6-31G**, and B3LYP/3-21G) can accurately describe all of the structural parameters with respect to their magnitude and sign when compared with the experimental values.

Meanwhile, in the semiempirical (AM1, PM3, and ZINDO) and Hartree-Fock (HF/6-31G, HF/6-31G*, HF/6-31G**, HF/3-21G, HF/3-21G*, HF/3-21G**, and HF/6-311G) methods there is not good agreement between the experimental and theoretical values for the torsion angles, especially the angle formed by atoms O2C3O13C12, with deviations −6.070° (AM1), −16.700° (PM3), −0.370° (ZINDO), +2.610° (HF/6-31G), +4.966° (HF/6-31G*), +4.900° (HF/6-31G**), +3.700° (HF/3-21G), +3.640° (HF/3-21G*), +3.820° (HF/3-21G**), +2.990° (HF/6-311G), +1.030° (B3LYP/6-31G), +3.200° (B3LYP/6-31G*), +3.220° (B3LYP/6-31G**) and +2.250° (B3LYP/3-21G) and exhibited standard deviations of 4.776 (AM1), 8.388 (PM3), 4.372 (ZINDO), 1.663 (HF/6-31G), 2.484 (HF/6-31G*), 1.762 (HF/6-31G**), 1.722 (HF/3-21G), 1.714 (HF/3-21G*), 1.797 (HF/3-21G**), 1.658 (HF/6-311G), 0.843 (B3LYP/6-31G), 1.227 (B3LYP/6-31G*), 1.103 (B3LYP/6-31G**) and 1.915 (B3LYP/3-21G), respectively.

Table 1 shows that for artemisinin (compound 1) the B3LYP/6-31G, B3LYP/6-31G*, B3LYP/6-31G** basis sets show excellent results for bond length, bond angle and torsion angle compared to the experimental data. The B3LYP/6-31G method described geometrical parameters well, with values close to the experimental results. However, the minimum base 6-31G has several deficiencies; thus, a polarization function was included to improve upon this base (*i.e*., *p* orbitals represented by *). Thus, 6-31G* refers to basis set 6-31G with a polarization function for heavy atoms (*i.e*., atoms other than hydrogen), and 6-31G** refers to the inclusion of a polarization function for hydrogen and helium atoms [29,30,31,32,33,34,35]. 

When basis sets with polarization functions are used in calculations involving anions, good results are not obtained due to the electronic cloud of anionic systems, which tend to expand. Thus, appropriate diffuse functions must be included because they allow for a greater orbital occupancy in a given region of space. It then becomes necessary to include diffuse functions in the basis function associated with the configuration of a neutral metal atom to obtain a better description of the metal complex. The 6-31G** basis is particularly useful in the case of hydrogen bonds [30,31,32,33,34,35].

Cristino *et al.* [36] used the B3LYP/6-31G* method to model artemisinin and 19 10-substituted deoxoartemisinin derivatives, with different degrees of activity against the *Plasmodium falciparum* D-6 strains of Sierra Leone. Chemometric methods (PCA, HCA, KNN, SIMCA, and SDA) were employed to reduce the dimensionality and to determine which subset of descriptors is responsible for the classification between more and less active agents.

Figueiredo *et al.* [37] conducted studies using the B3LYP/6-31G* method for antimalarial compounds against *Plasmodium falciparum* K1. These studies led to multivariate models for artemisinin derivatives and series of dispiro-1,2,4-trioxolanes. The application of these models has enabled the prediction of activity for compounds designed without known biological activity. Moreover, a new series of antimalarial compounds is currently in the study phase.

Araújo *et al.* [38] used density functional theory (6-31G*) to verify the performance of a base set in reproducing experimental data, particularly geometrical parameters, and to calculate the interaction energies, electronic states, and geometrical arrangements for complexes composed of a heme group and artemisinin. The results demonstrated that the interaction between artemisinin and the heme group occurs at long distances through a complex in which the iron atom of the heme group retains its electronic characteristics, with the quintet state being the most stable. These results suggest that the interaction between artemisinin and heme is thermodynamically favorable.

Pereira *et al.* [39] studied four structures of artemisinin by reductive decomposition A, B1, B2, and B3 with 13 species (QHS, 1/2, 3, 4, 5, 5a, 6, 7, 18, 18a, 19, 20, and 21), and the structures of the studied species were analyzed in terms of geometrical parameters, Löwdin bond orders, atomic partial charges, spin densities, electronic energies, free energies, and entropy. These studies were carried out at the B3LYP/6-31G** level.

Carvalho *et al.* [40] used the B3LYP/6-31G** method to study artemisinin and 31 analogues with antileishmanicidal activity against *Leishmania* *donovani*. The authors proposed a set of 13 artemisinins, seven of which are less active and six of which that have not been tested; of these six, one is expected to be more active against *L.* *donovani.*

Barbosa *et al.* [41] performed molecular modeling and chemometric studies involving artemisinin and 28 derivatives exhibiting anticancer activity and the calculations of the compounds studied were performed at the B3LYP/6-31G** level.

By comparing these methods with the DFT method (see Table 1), we find that all of the basis sets (B3LYP/6-31G, B3LYP/6-31G*, and B3LYP/6-31G**) have low standard deviations in relation to the semiempirical and Hartree-Fock methods at 0.843 (B3LYP/6-31G), 1.227 (B3LYP/6-31G*), and 1.103 (B3LYP/6-31G**). The variation was ±0.384 between B3LYP/6-31G and B3LYP/6-31G*, ±0.260 between B3LYP/6-31G and B3LYP/6-31G**, and ±0.124 between B3LYP/6-31G* and B3LYP/6-31G**. This study highlighted the B3LYP/6-31G** basis set, which is closer to the experimental results and shows good performance in the description when comparing the O2C3 and C3O13 bond length, O1O2C3 and C3O13C12 bond angles. The torsion angles or dihedral angle also showed good agreement with the experimental values reported in the literature, showing that with the 6-31G** basis set, the torsion angles O13C12C12aO1 and C12aO1O2C3 are closer to the artemisinin crystallographic data.

### 2.2. Principal Component Analysis (PCA) Results

The PCA results showed that the most important descriptors were the following: ALOGPS_logs, Mor29m, IC5 and GAP energy. They were chosen from the complete data set (1716 descriptors) and other variables were not selected because either they had a poor linear correlation with activity or they did not give a distinct separation between the more and less active.

The values of the important descriptors of each selected compound identified via PCA as well as the values of log*RA*, relative activity (RA) and the *IC*_50_ is the 50% inhibitory concentration are shown in Table 2. The Table 2 shows the Pearson correlation matrix between the descriptors and log*RA*, and the correlation between pairs of descriptors is less than 0.2420, while the correlation between the descriptors and log*RA* is less than 0.7459. The descriptors selected by PCA represent the characteristics necessary to separate between the more and less active with anticancer activity of these compounds against human hepatocellular carcinoma HepG2.

**Table 2:** Physicochemical properties selected by PCA, experimental log*RA* values, *IC*_50_ and the correlation matrix.

Compounds	ALOGPS_*log*	Mor29m	IC5	Gap Energy	log*RA*	*RA*	*IC*_50_/µΜ
1^-^	−2.3500	−0.3050	4.8620	0.2616	0.0000	1.0000	97
2^-^	−3.5200	−0.3070	5.2530	0.2525	−0.0132	0.9700	100
3^+^	−6.3500	−0.4550	5.6840	0.2521	1.5396	34.6417	2.8
4^+^	−6.8400	−0.5250	5.6240	0.2524	1.9075	80.8164	1.2
5^+^	−7.1600	−0.5140	5.5010	0.2527	2.3240	210.8628	0.46
6^+^	−7.4900	−0.5010	5.2250	0.2525	1.3635	23.0940	4.2
7^-^	−3.6400	−0.2360	5.2170	0.2467	−0.0132	0.9700	100
8^+^	−7.0300	−0.5260	5.5970	0.2462	2.1294	134.7100	0.72
9^-^	−7.6800	−0.1790	5.1970	0.2462	−0.0132	0.9700	100
10^-^	−3.6800	−0.3650	5.2530	0.2367	−0.0132	0.9700	100
11^-^	−3.6800	−0.3050	5.2530	0.2359	−0.0132	0.9700	100
12^+^	−6.9700	−0.3940	5.5080	0.2457	1.5396	34.6417	2.8
13^+^	−6.9700	−0.2910	5.5080	0.2552	1.3433	22.0444	4.4
14^-^	−7.4000	−0.2280	5.1590	0.2217	−0.0132	0.9700	100
15^-^	−7.4000	−0.2280	5.1590	0.2287	−0.0132	0.9700	100
16^-^	−3.7500	−0.4430	5.1800	0.2194	−0.0132	0.9700	100
17^-^	−7.6100	−0.3330	5.1680	0.2177	−0.0132	0.9700	100
18^+^	−5.4900	−0.3470	5.6380	0.2199	0.3604	2.2929	42.3
19^+^	−6.7200	−0.5520	5.5430	0.2491	1.8728	74.6105	1.3
20^+^	−7.0600	−0.5520	5.4190	0.2492	2.1002	125.9505	0.77
21^+^	−6.8400	−0.5150	5.5160	0.2449	1.4185	26.2119	3.7
**ALOGPS_ *log***		0.2420	−0.4260	0.0497	−0.5265	-	-
**Mor29m**			−0.5892	−0.2971	−0.8249	-	-
**IC5**				0.1767	0.7459	-	-
**Gap energy**					0.5238	-	-

The results of the PCA model are presented in Table 3. The model was constructed with three main components (3 PCs). The first principal component (PC1) describes 38.6537% of the total information, the second principal component (PC2) describes 21.5859%, and the third (PC3) 12.3501%. PC1 contains 48.3171% of the original data, and the combination of the first two components (PC1 + PC2) contains 75.2996%, and all three (PC1 + PC2 + PC3) explain 90.7373% of the total information, losing only 9.2627% of the original information. The descriptors ALOGPS_logs (0.4232), Mor29m (0.5937) and IC5 (−0.6223) contribute the most to PC1, while in PC2, the descriptor GAP energy (0.7746) is the primary contributor. The main components can be written as a linear combination of the selected descriptors. Mathematical expressions for PC1 (1) and PC2 (2) are shown below:

PC1 = 0.4232ALOGPS_log + 0.5937Mor29m − 0.6223IC5 − 0.2845Gap energy
(1)

PC2 = 0.5936ALOGPS_log − 0.1803Mor29m − 0.1225IC5 + 0.7746Gap energy
(2)

**Table 3:** PCA and contribution of selected descriptors based on step multivariate analysis.

Parameters	Main Component		
	PC1	PC2	PC3
Variance (%)	38.6537	21.5859	12.3501
Cumulative variance (%)	48.3171	75.2996	90.7373
Molecular descriptors		**Contribution**	
		**PC1**	**PC2**
ALOGPS_*log*		0.4232	0.5936
Mor29m		0.5937	−0.1803
IC5		−0.6223	−0.1225
Gap energy		−0.2845	0.7746

**Figure 2:**
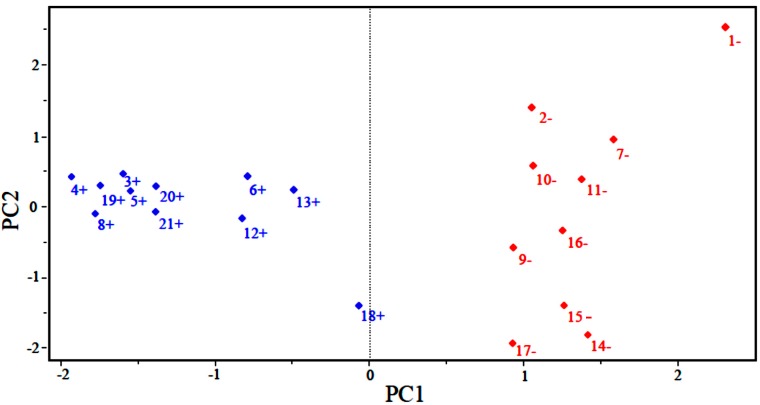
Plot of PC1–PC2 scores for artemisinin and derivatives with anticancer activity against human hepatocellular carcinoma HepG2 line. Positive values indicate more potent analogs (in blue), and negative values indicate less potent analogs (in red).

Figure 2 shows the scores for the 21 compounds studied. Based on the graph, PC1 distinguishes between compounds that are more potent and less potent. The most potent compounds are located at the left (3, 4, 5, 6, 8, 12, 13, 18, 19, 20 and 21), while the less potent compounds are located in the right side of the graph (1, 2, 7, 9, 10, 11, 14, 15, 16 and 17).

**Figure 3:**
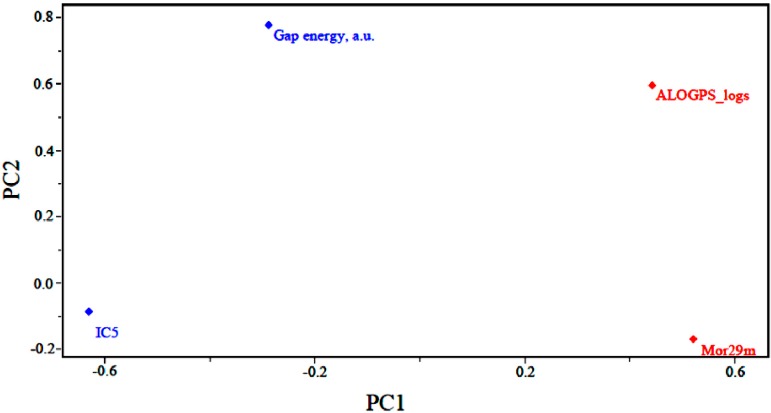
Plot of the PC1–PC2 loadings with the four descriptors selected to build the PLS and PCR models of artemisinin and derivatives with biological activity against human hepatocellular carcinoma HepG2 line.

Figure 3 shows the loadings for the four (4) descriptors that are most important in the classification of compounds. Less potent compounds have high contributions from the descriptors ALOGPS_logs and Mor29m, while more potent compounds have a high contribution from the descriptor GAP energy and IC5. Thus, the descriptors GAP energy and IC5 are responsible for the location of more potent compounds at the left side of the graph. The descriptors ALOGPS_logs and Mor29m places less potent compounds in the right part of the graph. Figure 3 also shows that the higher the contribution of the descriptors ALOGPS_logs and Mor29m in the first principal component, *i.e.*, the higher the value for a certain compound, the higher the score value will be, indicating that the compound is less potent than others. The other descriptors contribute to a lesser degree. For example, the descriptor GAP energy has negative weight in PC1, demonstrating that the most potent compounds generally have lower values of this descriptor.

### 2.3. Hierarchical Cluster Analysis (HCA) Results

The HCA method classified the compounds into two classes (more active and less active) and was based on the Euclidean distance and the incremental method [42]. In the incremental linkage, the distance between two clusters is the maximum distance between a variable in one cluster and a variable in the other cluster. The descriptors employed to perform HCA were the same as those used for PCA, *i.e*., ALOGPS_logs, Mor29m, IC5 and GAP energy.

**Figure 4:**
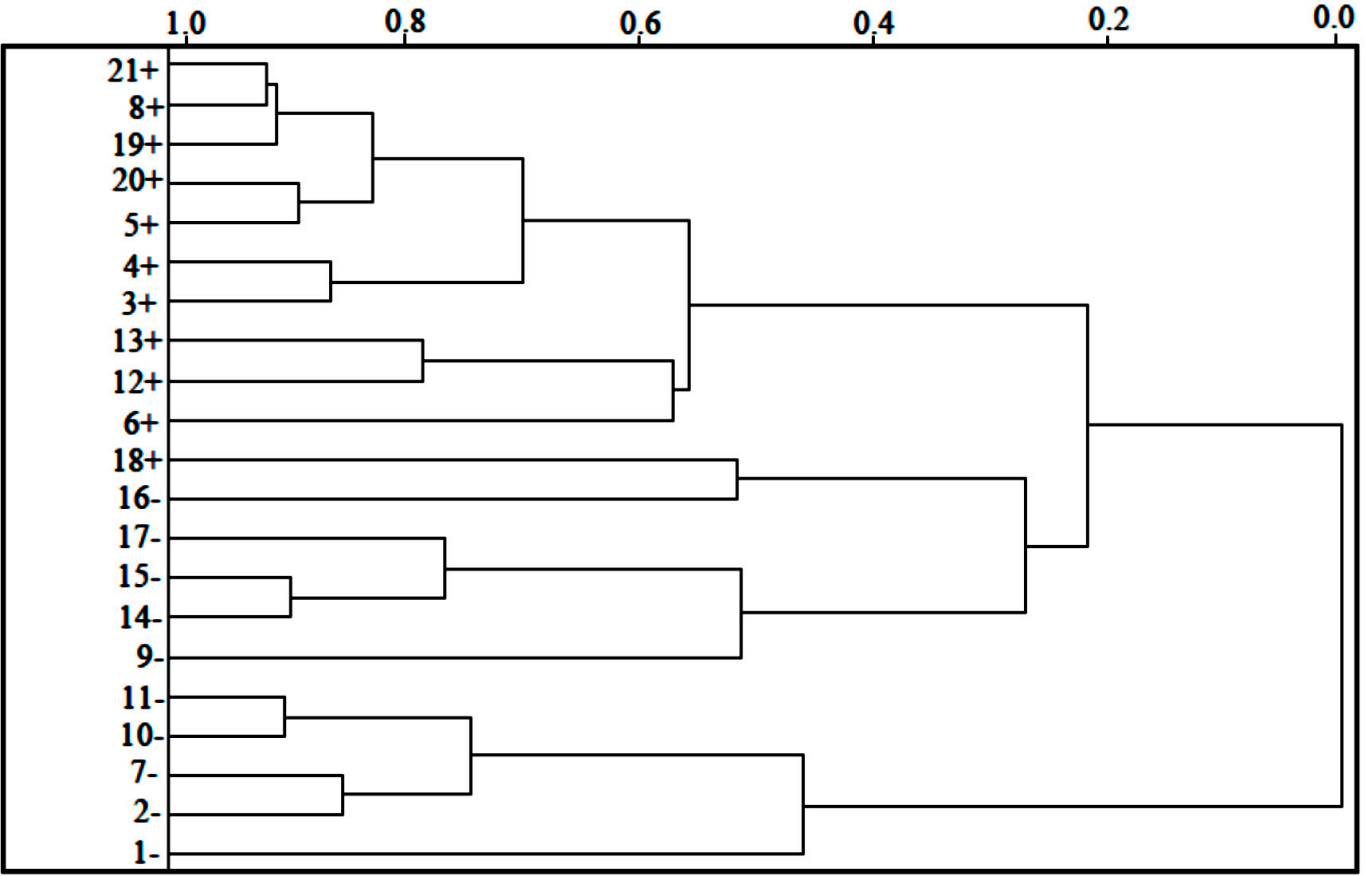
HCA dendrogram for artemisinin and derivatives with anticancer activity against human hepatocellular carcinoma HepG2*.* Positive values indicate more potent analogs, and negative values indicate less active compounds.

In the HCA technique, the distances between pairs of samples are computed and compared. Small distances imply that compounds are similar, while dissimilar samples will be separated by relatively large distances. The dendrogram in Figure 4 shows the HCA graphic as well as the compounds separated into two main classes. The scale of similarity varies from 0 for samples with no similarity to 1 for samples with identical similarity. By analyzing the dendrogram, some conclusions can be drawn even though the compounds present some structural diversity.

HCA showed results similar to those obtained with PCA. The compounds are grouped according to their biological activities. The most potent compounds are 3, 4, 5, 6, 8, 12, 13, 18, 19, 20 and 21. The less potent compounds are 1, 2, 7, 9, 10, 11, 14, 15, 16 and 17. Compound 18 has the lowest value of log*RA* = 0.3604, among the compounds classified as most potent of the series studied. Whereas, the compound 5 has the highest value of log*RA* = 2.3240, whereas the variation between the activities of the compounds 5 and 18 is ±1.9636 between them.

### 2.4. Partial Least Squares (PLS) and Principal Component Regression (PCR) Results

The statistical quality [43] of the PLS and PCR models was gauged by parameters such as correlation coefficient or squared correlation coefficient (R^2^), explained variance (R^2^_ajust_, *i.e.*, adjusted R^2^), standard deviation (s), variance ratio (F—a statistic of assessing the overall significance), cross-validated correlation coefficient (*Q*^2^), standard error of validation (*SEV*), predicted residual error sum of squares (*PRESS*) and standard deviation of cross-validation (*S_PRESS_*) [44,45,46]. The best regression models were selected based on high values of R^2^, R^2^_ajust_, Q^2^ and F and low values of s, *SEV*, *PRESS* and *S*_press_.

The calculated properties and the experimental activity values for the compounds studied were used to build the PLS and PCR regression models (see Table 4). The models built using the PLS and PCR were based on three latent variables and 21 compounds.

The regression equations obtained for PLS (Equation (3)) and PCR (Equation (4)) models that relate the descriptors and anticancer activity are the following:

log*RA* = −0.2748ALOGPS_log − 0.4307Mor29m + 0.3894IC5 + 0.2734 Gap energy (3)

*n* = 21, R^2^ = 0.9473, R^2^_ajust_ = 0.9381, s = 0.2280, F_(4,17)_ = 71.9013, *Q^2^* = 0.9151, *SEV* = 0.2620, *PRESS* = 0.8937, *S_PRESS_* = 0.0590.

log*RA* = −02904ALOGPS_log − 0.4074Mor29m + 0.4270IC5 + 0.1953 Gap energy (4)

*n* = 21, R^2^ = 0.9367, R^2^_ajust_ = 0.9256, s = 0.2514, F_(4,17)_ = 59.1211, *Q^2^* = 0.9063, *SEV* = 0.2752, *PRESS* = 1.0745, *S_PRESS_* = 0.0647.

The results obtained with the PLS and PCR models were very close, with variation between PLS and PCR of R^2^ = ±0.0106, R^2^_ajust_ = ±0.0125, s = ±0.0234, F_(4,11)_ = ±12.7802, Q^2^ = ±0.0088, SEV = ±0.0132, PRESS = ±0.4808 and S_PRESS_ = ±0.0057. The quality of the PLS and PCR models can be demonstrated by comparing the measured and the predicted activities. The validation errors obtained by the leave-one-out cross-validation method are shown in Table 4. For the PLS model, only six compounds (1, 3, 5, 18, 20 and 21) had high validation errors, and the PCR model yielded seven compounds (1, 3, 4, 5, 17, 18 and 20) with high residual values.

**Table 4:** Predicted PLS and PCR results and validation errors for log*RA* (experimental).

Compounds	Predicted		Validation Error		Experimental
	PLS	PCR	PLS	PCR	log*RA*
**1**^−^	−0.4002	−0.3420	−0.4002	−0.3420	0.0000
**2**^−^	0.3129	0.2298	0.3161	0.2166	−0.0132
**3**^+^	1.9110	1.8824	0.3714	0.3428	1.5396
**4**^+^	2.0905	2.0404	0.1830	1.1329	1.9075
**5**^+^	1.8148	1.7574	−0.5092	−0.5666	2.3240
**6**^+^	1.4038	1.3075	0.0403	−0.0560	1.3635
**7**^−^	−0.1312	−0.1548	−0.1444	−0.1680	−0.0132
**8**^+^	1.9071	1.9093	−0.2223	−0.2201	2.1294
**9**^−^	0.2824	0.2716	0.2692	0.2584	−0.0132
**10**^−^	0.1883	0.1772	0.1751	0.1640	−0.0132
**11**^−^	−0.0429	−0.0270	−0.0561	−0.0402	−0.0132
**12**^+^	1.3212	1.3357	−0.2184	−0.2039	1.5396
**13**^+^	1.1437	1.1276	−0.1996	−0.2157	1.3433
**14**^−^	−0.1448	0.0796	−0.1580	0.0664	−0.0132
**15**^−^	0.0023	0.1410	−0.0109	0.1278	−0.0132
**16**^−^	0.0131	0.1077	0.0001	0.0945	−0.0132
**17**^−^	0.1968	0.3439	0.1836	0.3307	−0.0132
**18**^+^	0.7639	0.8522	0.4035	0.4918	0.3604
**19**^+^	1.9530	1.9139	0.0802	0.0411	1.8728
**20**^+^	1.7459	1.6991	−0.3543	−0.4011	2.1002
**21**^+^	1.7443	1.7392	0.3258	0.3207	1.4185

**Figure 5:**
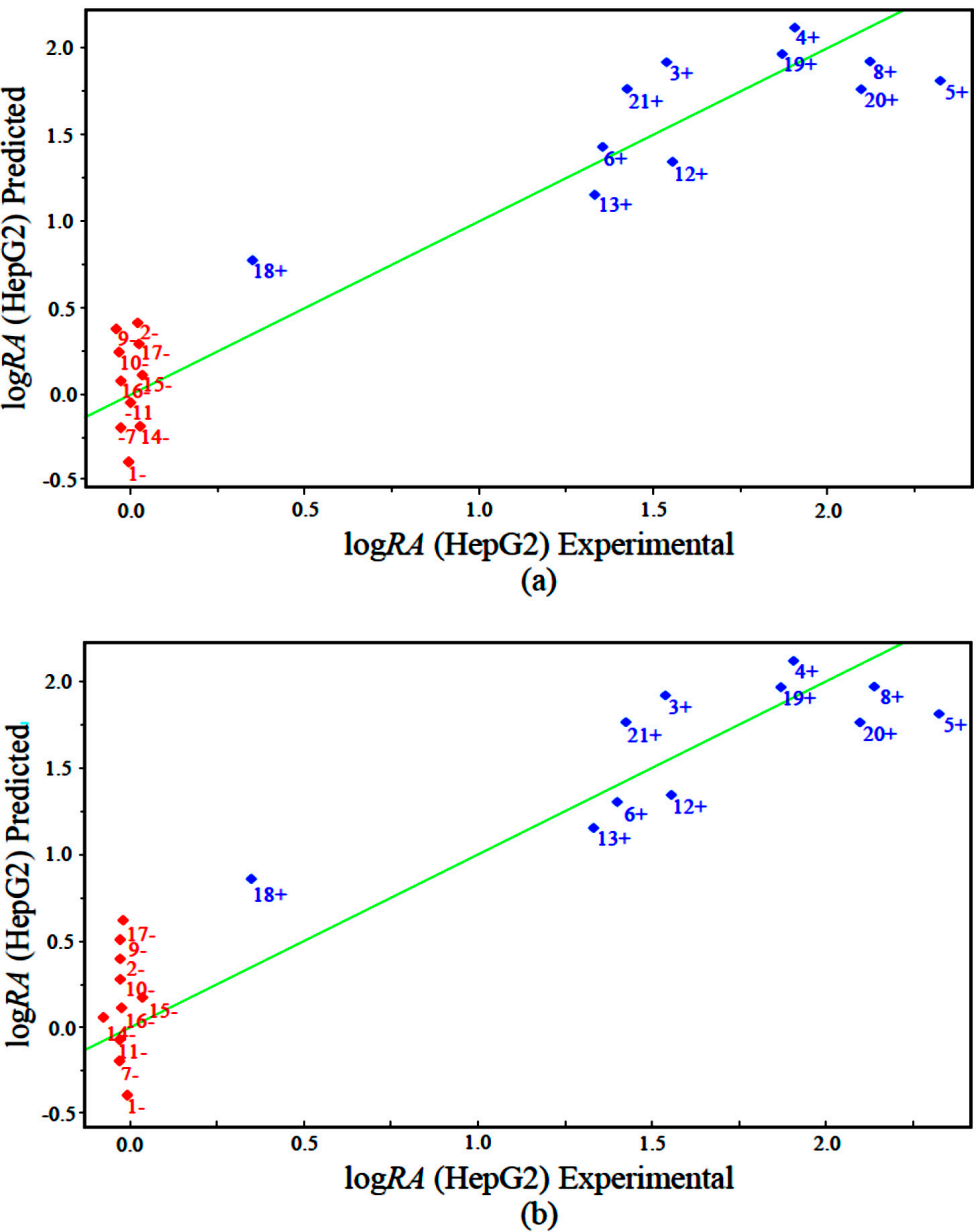
Plot of experimental *versus* predicted values for log*RA* modeled by (**a**) PLS and (**b**) PCR.

The measured versus predicted values using our PLS and PCR models are presented in Figure 5a,b, respectively. The PLS and PCR plots identify compounds with higher activity (blue) and compounds with lower activity (red). According to the PLS and PCR models, the four variables present different magnitudes of regression coefficients (in absolute value). The models reveal that compounds with high biological potency against human hepatocellular carcinoma HepG2 have a combination of higher values of IC5 and GAP energy and lower values of ALOGPS_logs and Mor29m for the PLS and PCR models.

The eight compounds of the test set (22–29) were molded from the most stable structure of artemisinin, compound 1 of Figure 1, and constructed using GaussView 5.0 program, carrying the complete optimization of the geometry of each compound with the basis set of separated valence B3LYP/6-31G** using the DFT method as implemented in Gaussian 03 program. After obtain the most stable geometry of each compound was determined only selected descriptors in PCA and used in the construction of the QSAR models, namely ALOGPS_logs, Mor29m, IC5 and GAP energy, shown in Table 5.

**Table 5:** Molecular properties selected by analysis of main components of test set with anticancer activity unknown.

Test Set	ALOGPS_*log*	Mor29m	IC5	Gap energy, a.u.
**22**	−5.030000	−0.412000	5.514000	0.252200
**23**	−5.760000	−0.443000	5.628000	0.252200
**24**	−7.390000	−0.515000	5.364000	0.252400
**25**	−7.140100	−0.305100	5.571100	0.219700
**26**	−6.030000	−0.311000	5.572000	0.252400
**27**	−4.820000	−0.518000	5.856000	0.251700
**28**	−7.350000	−0.601000	5.280000	0.227600
**29**	−7.010000	−0.543000	5.488000	0.232300

**Figure 6:**
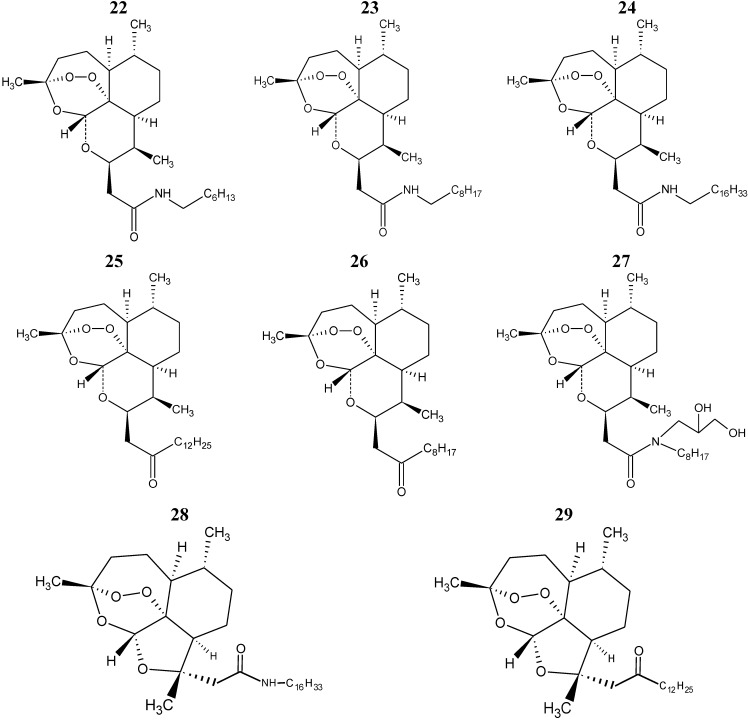
Compounds of the test set artemisinin derivatives with unknown anticancer activity against human hepatocellular carcinoma HepG2.

The QSAR models (PLS and PCR) were built used to predict the unknown anticancer activity of eight new artemisinin derivatives shown in Figure 6, compounds 22–29. Table 6 shows the results of the log*RA* by PCR and PLS models. According to Table 6 the PLS and PCR models showed that all the compounds of the test set are predicted to be more active, they had values of log*RA* greater than zero (log*RA* > 0) in both models (PLS and PCR) with residues of prediction ranging from 0.0650 to −0.0560, suggesting that these new compounds in the two models (PLS and PCR) are more potent than artemisinin may be synthesized and tested for anticancer activity.

**Table 6:** Anticancer activity predicted (log*RA*) by PCR and PLS models for the test set compounds and residues of prediction between models.

Test Set Compounds	Predicted (log*RA*)		Residues of Prediction (PLS-PCR)
	PLS	PCR	
**22**	1.2458	1.2048	0.0410
**23**	1.6431	1.6210	0.0221
**24**	1.6804	1.6154	0.0650
**25**	0.6841	0.8649	−0.1808
**26**	1.1631	1.1564	0.0067
**27**	2.1201	2.1163	0.0038
**28**	1.3444	1.3850	−0.0406
**29**	1.5410	1.5970	−0.0560

### 2.5. Pharmacokinetic and Toxicological Results

The prediction of Absorption, Distribution, Metabolism and Excretion (ADME) proprieties for artemisinin and its derivatives of the test set (compounds 22–29) classified by PLS and PCR models as more potent are shown in Table 7 and Table 8. In Table 7, one can observe the absorption values (HIA, PCaCO2 and PMDCK) predicted for the compounds. The prediction of human intestinal absorption is a major objective in the optimization and selection of candidates for the development of oral medications. The focus on the discovery of modern drugs is not simply in the pharmacological activity, but also in search of more favorable pharmacokinetic properties [47]. The results of human intestinal absorption are the sum of absorption and bioavailability, evaluated from the proportion of excretion or cumulative excretion in urine, bile and feces [48,49].

The test compounds showed good human intestinal absorption, having values of HIA > 90%, being close to that of artemisinin (compound 1). Compound 27 showed the lowest absorption equal to 94.2039%, whereas compound 26 showed the highest value of HIA equal to 98.1189%, as shown in Table 7.

The P_Caco2_ (nm/s) and P_MDCK_ (nm/s) cell models have been used as a reliable *in vitro* model for the prediction of oral drug absorption, being the Caco-2 cells derived from human colon adenocarcinoma and have various routes of drug transport through the intestinal epithelium [49]. The results of the compounds shown in Table 7 showed an average permeability of 45.4351, as proposed by Yazdanian [50]. The values obtained of P_CaCO2_ (nm/s) were higher than 30.3276 nm/s (compound 1, artemisinin). The compounds 25 and 26 showed higher values of cell permeability of 51.2476 and 51.5452 nm/s, respectively.

**Table 7:** Absorption properties for artemisinin (compound 1) and compounds of the test set.

Compounds	Absorption			
	HIA(%) ^[a]^	P_CaCO2_(nm/s) ^[b]^	P_MDCK_(nm/s) ^[c]^	P_skin_^[d]^
1	96.3143	30.3276	72.4627	−3.00248
22	95.9522	48.074	0.2820	−2.78573
23	96.0180	49.0102	2.7481	−2.38535
24	96.1170	50.8969	64.4258	−1.10239
25	97.6636	51.2473	54.1962	−1.00477
26	98.1189	51.5452	13.6801	−1.4846
27	94.2039	35.0362	0.0437	−2.66011
28	96.1170	46.0453	64.766	−0.792156
29	97.6636	46.7337	55.4025	−0.768943

^[a]^: percentage of human intestinal absorption; ^[b]^: cell permeability (Caco-2 in nm/s); ^[c]^: cell permeability Maden Darby Canine Kidney in nm/s; ^[d]^: skin permeability.

**Table 8:** Distribution properties in percentages of PPB and penetration of the blood brain barrier for artemisinin (compound 1) and compounds of the test set.

Compounds	Distribution	
	PPB(%) ^[a]^	C_Brain_/C_Blood_^[b]^
1	93.368123	1.30488
22	90.481620	3.1575
23	91.279366	5.35648
24	93.306402	11.0801
25	96.696312	8.39023
26	95.399268	2.65831
27	90.056670	1.91129
28	93.838777	10.9862
29	97.347576	8.08563

^[a]^: percentage of plasma protein binding; ^[b]^ penetration of the blood brain barrier.

In accordance with Irvine *et al.* [51], P_MDCK_ (nm/s) system cells can be used as tool for rapid screening permeability. The test compounds (22, 23, 26 and 27) were those that presented low permeability in the P_MDCK_ (<25) cell system. In the studied set, compounds 22 and 27 showed the lowest permeability values P_MDCK_ equal to 0.2820 and 0.0437 nm/s, respectively. Compounds 24, 25, 28 and 29 showed the highest permeability values varying in the range from 54.1962 to 64.7660 nm/s, close to the permeability value of artemisinin.

In the pharmaceutical, cosmetic and agrochemical industries, predicting the rate of skin permeability is a crucial parameter for transdermal administration of medications and for the risk assessment of chemical products that come into contact with the skin accidentally [52]. The test set compounds showed negative values of skin permeability, *i.e*, it is not important to be administered for transdermal use, and also not present any risk accordance results described in Table 7.

The distribution of a drug depends on its plasma protein binding (PPB) and partition in adipose tissue and other tissues. In plasma the drug may be in unbound or bound form, which depends on the affinity that the drug presents by the plasmatic protein (drug target). If the protein binding is reversible, then a chemical equilibrium will exist between bound and unbound states. The proteins binding can influence in the biological half-life in the body. The bound portion may act as a reservoir or deposit to which the drug is slowly released in the unbound form. As the non-bound form being metabolized and/or excreted from the body, fraction bound to will be released in order to that maintain balance [53,54]. In Table 8 shows the results of the distribution properties (PPB% and C_Brain_/C_Blood_) for artemisinin and classified as most potent compounds of test set. Compounds 22–29 showed strong plasma protein binding with PPB > 90.0566%, being close to the value of PPB of artemisinin which was equal to 93.3681%. Compounds 25, 26 and 29 showed higher strength in plasma protein binding equal to 96.6963%, 95.3992% and 97.3475%, respectively.

The penetration of the blood brain barrier is critical in the pharmaceutical field, because compounds that act on the central nervous system (CNS) should go through it, and inactive compounds in CNS should not go in order to avoid collateral effects of CNS [55]. In the test set, all compounds showed absorption values to the CNS higher than 1, and in accordance with the classification proposed by Ma *et al.* [56], compounds that have values greater than 1 (C_Brain_/C_Blood_ > 1) are classified as active in the CNS may cause collateral effects, and compounds that have values below 1 (C_Brain_/C_Blood_ < 1) are classified as inactive in the CNS. Therefore, compounds 22–29 had a variation of C_Brain_/C_Blood_ in relation to the artemisinin of 1.8526, 4.0516, 9.7752, 7.0853, 1.3534, 0.6064 and 9.6813, respectively. Since the compound 27 showed the value of penetration of the blood brain barrier (C_Brain_/C_Blood_) closest to of artemisinin (C_Brain_/C_Blood_ = 1.304) having the smallest variation between test compounds studied (C_Brain_/C_Blood_[compound 27] − C_Brain_/C_Blood_[artemisinin]), showing value equal to 0.6064.

Table 9 shows the results of the toxicological properties of mutagenicity (Ames Test) and carcinogenicity (Mouse and rat) for artemisinin and its derivatives of the test set (22–29) classified by PLS and PCR models as more potent with anticancer activity against human hepatocellular carcinoma HepG2. One of the important reasons for the discovery of new drugs is the evaluation of the toxicity of drug candidates. This means that the conception of drugs with consideration of its toxicity is very important, as well as predicts the mutagenicity and carcinogenicity of new compounds that may be toxic.

**Table 9:** Toxicological properties of mutagenicity (Ames Test) and carcinogenicity (mouse and rat) for artemisinin and its derivatives of the test set (22–29).

Compounds	Ames Test	Carcinogenicity	
	Mutagenicity	Mouse	Rat
1	Mutagenic	Negative	Positive
22	Non-mutagenic	Negative	Positive
23	Non-mutagenic	Negative	Positive
24	Non-mutagenic	Negative	Positive
25	Non-mutagenic	Positive	Positive
26	Non-mutagenic	Positive	Positive
27	Non-mutagenic	Negative	Negative
28	Non-mutagenic	Negative	Positive
29	Non-mutagenic	Negative	Positive

The Ames test is a simple method to test mutagenicity of a compound, suggested by Ames, where various strains of *Salmonella typhimurium* bacterium with mutations in the genes involved in histidine synthesis, so they require histidine for growth, are used. The variable being tested is the ability of the mutagenic agent to provoke the reversal of the growth in histidine-exempt medium [57]. In this method, compound 1 (artemisinin) presented positive prediction, which means that this compound was predicted as a mutagen. The other compounds (22–29) showed a negative prediction, ie, were predicted as non-mutagenic, as shown in Table 9.

Carcinogenicity is the ability that a substance has to induce alterations that lead to cancer. The carcinogenicity assays require a long time (>2 years). The principal methodologies use “*in vivo*” assays, using mice or rats by exposing them to a chemical compound, where the observed variable is the existence of cancer. In this study, PreADMET server was used to predict the result which is constructed from the data of the NTP (National Toxicology Program) and the USA/FDA, which are the results of *in vivo* tests for carcinogenicity in mice and rats for 2 years.

In the prediction of carcinogenicity in mouse, compounds 25 and 26 showed positive prediction, ie, no evidence of carcinogenic activity. The others compounds were predicted as negative, which means that there is evidence of carcinogenic activities in mouse, for such compounds (1, 22–24 and 27–29). In the prediction of carcinogenicity in rat, the following compounds 1, 22–26, 28 and 29 had positive prediction, demonstrating that show no carcinogenic activity. Whereas compound 27 showed negative prediction, meaning that this compound may exhibit carcinogenic activity.

## 3. Experimental Section

### 3.1. Anticancer Compounds Studied

Initially, 21 artemisinins (artemisinin and its derivatives) with different degrees of cytotoxicities against human hepatocellular carcinoma HepG2 were selected from the literature (Figure 1) [24]. The employed strategy was based on the knowledge that the endoperoxide group presented in artemisinin and its derivatives is responsible for their antimalarial, antileishmanicidal and anticancer activities. The compounds, the subjects of this study, consisted of artemisinin, amides, esters, alcohols, ketones, derivatives with polar hydroxyl and carboxylic acid groups and five-membered ring derivatives. All compounds have been associated with *in vitro* bioactivity against a human hepatocellular carcinoma cell line, HepG2.

The numbering of the atoms used in this study is shown in Figure 1 (compound 1—artemisinin). The logarithm of the *IC_50_* value of artemisinin over the *IC_50_* value of the compounds (logarithm of relative activity, log*RA*) was used to reduce inconsistencies caused by individual experimental environments:

log*RA* = log(*IC_50_* of artemisinin/*IC_50_* of analog) (5)

where *IC_50_* is the 50% inhibitory concentration. In this study, the following classification based on the anticancer responses was adopted: compounds with log*RA* > 0.00, ranging from 0.3604 to 2.324, were assumed to be more potent analogs (3, 4, 5, 6, 8, 12, 13, 18, 19, 20 and 21), and those with log*RA* ≤ 0.00, ranging from 0.0000 to −0.0132, were considered to be less potent analogs (2, 7, 9, 10, 11 and 14–17). The compound 5 (log*RA* = 2.324) is the most potent compound in the series studied.

### 3.2. Molecular Modeling and Calculations of Descriptors or Properties Molecular

Molecular modeling started with the construction of the structure of artemisinin using the GaussView 3.0 program [58], which was then optimized with different methods and basis sets—semiempirical (AM1, PM3, and ZINDO), Hartree-Fock (HF/6-31G, HF/6-31G*, HF/6-31G**, HF/3-21G, HF/3-21G*, HF/3-21G**, and HF/6-311G), and DFT (B3LYP/6-31G, B3LYP/6-31G*, B3LYP/6-31G**, and B3LYP/3-21G).

These calculations were executed to find the method and basis sets with the best fit between the computational time and accuracy of the information compared to the experimental data [59]. After initial determination and structural optimization, the theoretical geometrical parameters of artemisinin in the region of the 1,2,13-trioxane ring (bond length, bond angle and torsion angle) were determined with the aim of evaluating the quality of the molecular wave function and standard deviation of method studied comparing the theoretical geometrical parameters with the experimental data (see Table 1).

The experimental structure of artemisinin was taken from the Cambridge Structural Database CSD, with REFCODES: QNGHSU10, crystallographic R factor 3.6 [60]. All the other structures (see Figure 1) were built with the optimized structure of artemisinin using the Gaussian 03 program [61] with the DFT method and B3LYP/6-31G** basis set. After the structures were determined in 3D, various descriptors for each molecule of the set studied were calculated.

The descriptors are important for the quantitative description of molecular structure and to finding appropriate predictive models [62]. The computation of the descriptors was performed employing the following software: Gaussian 03 program [61], e-Dragon [63,64], Molekel [65] and HyperChem 6.02 [66]. The e-Dragon program calculated 1666 descriptors that were divided into the following 20 classes: 48 constitutional descriptors; 47 descriptors of quantity and trajectory; 47 information indexes; 107 adjacency indexes; 21 topological charge indexes; 41 molecular Radic profiles; 150 RDF descriptors; 154 functional groups; 14 charge descriptors; 33 connectivity indexes; 96 2-D autocorrelations, 64 Burden eigenvalues; 44 indexes based on eigenvalues; 74 geometric descriptors; 160 MORSE-3D; 120 fragments centered in the atom; 31 molecular property descriptors; 119 topological indexes; 99 WHIM descriptors; and 197 Getaway descriptors. Other descriptors such as the following were obtained:

(a) QUANTUM CHEMICAL descriptors: In our study, we calculated the following 25 quantum-chemical descriptors: total energy (TE), energy of the highest occupied molecular orbital (HOMO), a level below the energy of the highest occupied molecular orbital (HOMO − 1), lowest unoccupied molecular orbital energy (LUMO), a level above the energy of the lowest unoccupied molecular orbital (LUMO + 1), difference in energy between HOMO and LUMO (GAP = HOMO − LUMO), Mulliken electronegativity (χ), molecular hardness (η), molecular softness (1/η), and charge on the atom n (where n = 1, 2, 3, 4, 5, 5a, 6, 7, 8, 8a, 9, 10, 11, 12, 12a, 13). The atomic charges used in this study were obtained with the key word POP = CHELPG using the electrostatic potential [67], with this strategy, it was possible to obtain the best potential molecular series of points defined around the molecule, and atomic charges offer the general advantage of being physically more satisfactory than Mulliken charges [68].

(b) Descriptors related to quantitative properties of chemical structure and biological activity: In our data matrix, QSAR descriptors were included, *i.e.*, total surface area (TSA), molecular volume (MV), molar refractivity (MR), molar polarizability (MP), coefficient of lipophilicity (logP), molecular mass (MM) and hydration energy (HE) according to the HyperChem 6.02 program. The molecular descriptors were selected to provide valuable information about the influence of electronic, steric, hydrophilic and hydrophobic features on the anticancer activity of artemisinins.

### 3.3. Variable Selection and Model Building QSAR (PLS and PCR)

After the determination of all molecular descriptors, it was possible to construct a data matrix to develop step multivariate analysis. The step multivariate analysis was necessary to make the autoscale or standardizing data matrix X = (*n, m*) consisting of twenty-one (21) lines (the anticancer compounds studied) and one thousand seven hundred sixteen (1,716) columns (in this case, the calculated descriptors for each molecule), where *n* is the number of compounds studied and *m* is the number of variables.

The aim of using the standardizing matrix is to give each variable equal weight in mathematical terms, so each variable was centered on the mean and scaled to unit variance. To reduce the data set, variables were selected based on the analysis of the correlation matrix between variables (descriptors) and the logarithm of the relative activity (log*RA*).

The descriptors with small or no correlation (under the 0.20 correlation value cutoff) were discarded, resulting in only two hundred and thirteen (213) descriptors remaining from the initial set of one thousand seven hundred sixteen (1,716) descriptors. After this data compression, two complementary methods for exploratory data analysis were employed (PCA and HCA) to study intersample and intervariable relationships and to select the properties that contribute the most to the classification of the compounds into two groups [27,28]. One group contained more potent analogs and the other less potent analogs. PCA was employed to reduce the dimensionality of the data, find descriptors that could be useful in characterizing the behavior of the compounds acting against a human hepatocellular carcinoma cell line (HepG2) and look for natural clustering in the data and outlier samples.

While performing PCA, several attempts to obtain a good classification of the compounds were made. At each attempt, the score and loading plots were analyzed based on the variables employed in the analysis. The score plot gives information about the compounds (similarity and differences). The loading plot gives information about the variables (how they are connected to each other and which best describe the variance in the original data) [27,28]. The descriptors selected by PCA were used to perform HCA, PLS and PCR.

The objective of HCA was to present the compounds distributed in natural groups and the results confirm the PCA results. Thus, several approaches were attempted to establish links between samples/cluster. All of them were of an agglomerative type because each sample was first defined as its own cluster, and then others were grouped together to form new clusters until all the samples were part of a single cluster [28].

The QSAR models for the new artemisinin compounds with anticancer activity were constructed by the PCR and PLS methods based on the autoscaled data and the leave-one-out crossvalidation procedure [25,26,27,28]. The final purpose of the multivariate analysis (PLS and PCR) was the construction of a mathematical model that can be used to predict anticancer activity of the compounds studied. The statistical parameters used to assess the quality of the models were the Prediction Residual Error Sum of Squares (PRESS), Equation (6), the Standard Error of Validation (SEV), Equation (7), the total variance explained, R^2^ (correlation between the estimated values predicted by the model built with the full data set and actual values of y), Q^2^ (the cross-validated correlation coefficient) and SPRESS (standard deviation of cross-validation) given by Equations (8)-(10), respectively [27,28,69,70,71]:

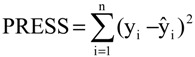
(6)

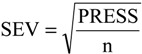
(7)

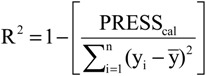
(8)

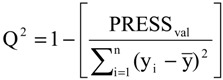
(9)

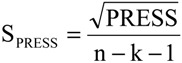
(10)


In Equations (6) and (7), n is the number of compounds used for the calibration or validation model, yi is the experimental value of the physicochemical property for the sample and ŷi is the value predicted by a calibration or validation model. In Equations (8) and (9), PRESScal is the Calibration Prediction Error Sum of Squares and PRESSval is the Validation Prediction Error Sum of Squares. Both PRESScal and PRESSval are evaluated from Equation (6) by changing ŷi for a calibration or validation model. The values of explained variance (R^2^_ajust_, *i.e.*, adjusted R^2^), standard deviation (s) and F (Fisher test) were determined. The multivariate data analyses (PCA, HCA, PLS and PCR) were performed by employing Pirouette 3.01 software [42].

### 3.4. Pharmacokinetic and Toxicological Properties of Test Compounds

At a molecular level, a system is coordinated by transporters, channels, receptors and enzymes; this system affects the absorption, distribution, metabolism, excretion and toxicity (ADME/Tox) of a molecule in humans. Understanding the interactions between small molecules and their molecular targets should improve the ability to predict the toxic consequences that are responsible for the removal of many commercialized drugs and failures in the final stage drug development [35,72,73,74].

Traditional ADME/Tox studies provide a detailed understanding of individual proteins, in which it is possible to examine if the molecule also binds to receptors that affect the regulation of other proteins, and if it interferes with endogenous metabolic, regulatory proteins and transport. Alternatively the main metabolic via may be mediated by a polymorphic enzyme and likely affect the therapeutic dose [73,75,76].

The properties ADME/Tox for artemisinin and its derivatives of the test set (22–29) were calculated using the server PreADMET [49]. This server calculates pharmacokinetic properties as: human intestinal absorption, cellular permeability Caco-2 *in vitro*, cell permeability Maden Darby Canine Kidney (MDCK), skin permeability, plasma protein binding and penetration of the blood-brain barrier, and toxicological properties as mutagenicity and carcinogenicity.

## 4. Conclusions

The DFT method and the B3LYP/6-31G** basis set revealed themselves to be adequate to optimize the structures of artemisinin and derivatives for subsequent study. The predictive classification models for artemisinin derivatives were obtained with a set of molecular descriptors selected by chemometric approaches. PCA and HCA methods classified the compounds studied into groups according to their degree of anticancer activity against a human hepatocellular carcinoma cell line (HepG2). The descriptors ALOGPS_logs, Mor29m, IC5 and GAP energy were responsible for distinguishing compounds with higher and lower anticancer activity. The molecular features represented by these descriptors are in good agreement with previous SAR analysis performed on artemisinin derivatives. The combination of these structural attributes is believed to govern the anticancer effects of the compounds studied in this work. The PLS and PCR models obtained here showed not only statistical significance but also predictive ability. The test set showed for two new artemisinin compounds satisfactory results for anticancer activity and pharmacokinetic and toxicological properties. Through this strategy and our findings, useful information was obtained that could be of use in experimental syntheses and biological evaluation to understand the molecular and structural requirements for designing new ligands to be used as anticancer agents. Consequently, further studies need be done to evaluate the different proposals as well as their actions, toxicity, and potential use for treatment of cancers.
